# Effects of *Moringa oleifera* Lam. Supplementation on Inflammatory and Cardiometabolic Markers in Subjects with Prediabetes

**DOI:** 10.3390/nu14091937

**Published:** 2022-05-05

**Authors:** Ligia E. Díaz-Prieto, Sonia Gómez-Martínez, Iván Vicente-Castro, Carlos Heredia, Elena A. González-Romero, María del Carmen Martín-Ridaura, Mercedes Ceinos, María J. Picón, Ascensión Marcos, Esther Nova

**Affiliations:** 1 Immunonutrition Research Group, Department of Metabolism and Nutrition, Institute of Food Science and Technology and Nutrition (ICTAN)—CSIC, 28040 Madrid, Spain; ldiaz@ictan.csic.es (L.E.D.-P.); sgomez@ictan.csic.es (S.G.-M.); i.vicente@ictan.csic.es (I.V.-C.); amarcos@ictan.csic.es (A.M.); 2 Cea Bermúdez Primary Health Care Centre, Madrid Health Service, 28003 Madrid, Spain; cheredia@salud.madrid.org (C.H.); elenaaurora.gonzalez@salud.madrid.org (E.A.G.-R.); 3 Madrid-Health, Madrid City Hall, 28007 Madrid, Spain; martinrmc@madrid.es (M.d.C.M.-R.); ceinosamm@madrid.es (M.C.); 4 Virgen de la Victoria University Hospital, 29010 Málaga, Spain; mjpiconcesar@gmail.com

**Keywords:** *Moringa oleifera* Lam., food supplement, prediabetes, glycemic control, inflammatory markers, serum lipid profile, blood pressure, total antioxidant capacity, fecal calprotectin

## Abstract

Different parts of the *Moringa oleifera* Lam. (MO) tree are consumed as food or food supplements for their nutritional and medicinal value; however, very few human studies have been published on the topic. The current work was aimed to provide ancillary analysis to the antidiabetic effects previously reported in a double-blind, randomized, placebo-controlled, parallel group intervention conducted in patients with prediabetes. Thus, the effect of MO leaves on blood and fecal inflammatory markers, serum lipid profile, plasma antioxidant capacity and blood pressure was studied in participants who consumed 6 × 400 mg capsule/day of MO dry leaf powder (MO, *n* = 31) or placebo (PLC, *n* = 34) over 12 weeks. Differences between groups were assessed using each biomarker’s change score with, adjustment for fat status and the baseline value. In addition, a decision tree analysis was performed to find individual characteristics influencing the glycemic response to MO supplementation. No differences in the biomarker’s change scores were found between the groups; however, the decision tree analysis revealed that plasma TNF-α was a significant predictor of the subject’s HbA1c response (improvement YES/NO; 77% correct classification) in the MO group. In conclusion, TNF-α seems to be a key factor to identify potential respondents to MO leaf powder.

## 1. Introduction

*Moringa oleifera* Lam. (MO) is a tree originally from Asia, grown in most tropical and subtropical areas and with cultivars recently introduced on the Mediterranean coast. MO leaves are nutritionally rich, as they contain high protein levels and abundant fiber, potassium, calcium, magnesium, β-carotene, α-tocopherol and polyphenols [1,2]. MO leaves as well as seeds and pods have been traditionally used as food or food supplements, with medicinal properties including antihypertensive, diuretic, antihyperlipidemic, antispasmodic, antiulcer, hepatoprotective, antidiabetic, antineoplastic, anti-inflammatory, antibacterial and antifungal activities [3,4,5]. Some of these bioactivities have been proven for a variety of leaf compounds, such as peptide fractions [6], isolated polysaccharides [7] and the isothiocyanate (ITC) derivatives of characteristic glucosinolates [8]. In this sense, moringin resulting from myrosinase-hydrolysis of glucomoringin under neutral conditions has been shown to exhibit effective antioxidant, anti-inflammatory and antitumor activities [9]. Other important phytochemicals in MO leaves are flavonoids and phenolic acids [10].

Regarding the antidiabetic effect of MO, a fairly large number of studies have been performed in animal models of hyperglycemia. Most of them show significant improvements in blood glucose, both fasting and in response to a glucose tolerance test [11]. Thus, the activity of MO as a natural antihypoglycemic agent and its potential application for diabetes prevention and treatment has gained considerable interest, since it is regarded as affordable and less prone to induce side effects than other pharmacological treatments [12]. However, only nine clinical trials in humans have been published [13,14,15,16,17,18,19,20,21]. The postprandial studies have shown significant or marginally [13] significant results both in patients with type 2 diabetes mellitus (DM) and healthy subjects [13,15,16], and all longitudinal studies except one [20] reported benefits on fasting blood glucose [17,19] or glycated hemoglobin [18] or both [21].

Prediabetes is characterized by above-normal values of glycemia, although lower than those used for DM diagnosis. Progression of this metabolic alteration is independently associated with abdominal obesity indicators as observed in a 4-year follow-up study [22]. In obesity, excessive adiposity, increased adipose tissue lipolysis, ectopic accumulation of circulating fatty acids in insulin sensitive tissues, insulin resistance and inflammation characterized by augmented production of inflammatory cytokines by macrophages infiltrating the adipose tissue and excessive reactive oxygen species are all related as etiology factors involved in DM development [23,24]. On this basis, plants and herbs such as MO, with the capacity to modify the transcriptional regulation of enzymes involved in lipid and glucose metabolism, can potentially influence the biomarkers associated with metabolic and cardiovascular health and inflammation in at-risk individuals. In this respect, MO has shown prevention of histopathological changes in the liver of diabetes-induced animals, with reduced lipogenic and increased lipolytic gene expressions in this organ and also a significant hypolipidemic effect [11,25,26]. Potent antioxidant and immunomodulatory actions, including an inhibitory effect on proinflammatory mediators such as inducible nitric oxide synthase (iNOS), cyclooxygenase (COX)-2, prostaglandin E (PGE)-2, tumor necrosis factor (TNF)-α and interleukin (IL)-1β and IL-6 have been evidenced in in vitro [27,28] and in vivo experiments [25,29,30]. Moringin is the most abundant MO isothiocyanate, with proven antioxidant and anti-inflammatory properties [31,32,33]. However, other compounds found in specific fractions that have shown inhibition of pro-inflammatory mediator production have been pointed out as potential bioactive molecules. In these sense, seven compounds were identified by liquid chromatography/mass spectrometry analysis of an ethyl acetate extract fraction of MO leaves [28]. Antihypertensive activity has also been attributed to MO extracts or peptide fractions [34,35]. Human studies on hypolipidemic effects [17,36] and the anti-inflammatory and antihypertensive activity [20,37] provide limited evidence due to the few number of published studies, their small sample size and the heterogeneity of the study designs. The effects observed in human studies are highly dependent on the dose and duration of the study and the basal condition of the study participants.

Our group recently published a clinical trial showing the beneficial effects of MO on the glycemic control of prediabetic patients [21]; as an ancillary purpose, the potential modifications in inflammatory markers, lipid profile, plasma antioxidant capacity and blood pressure of the studied participants were also assessed. The corresponding results are presented here. In addition, the influence of the baseline value of percentage body fat as well as inflammatory and cardiometabolic markers on the control of glycemia by MO supplementation was evaluated.

## 2. Materials and Methods

### 2.1. Study Design

The design and protocol of this nutritional intervention study has been published elsewhere [21] and can be consulted for information not reported here. A double-blind, randomized, placebo-controlled, parallel group study was conducted in patients with prediabetes. The intervention with 6 capsules containing 400 mg of MO dry leaf powder or placebo was carried out over 12 weeks. Eligible participants were randomized using a simple block randomization of 1:1. The study was registered in www.ClinicalTrials.gov (accessed on 4 May 2022) (Identifier: NCT04734132).

### 2.2. Study Participants

Subjects with prediabetes who had never used drugs for glycemic control and were within the age of 40 to 70 y were included. Prediabetes was diagnosed following the American Diabetic Association (ADA) criteria [38]: HbA1c: 5.7–6.4%, or fasting glucose between 100 and 125 mg/dL, or 2 h glucose tolerance test between 140 and 199 mg/dL. The recruitment procedures and exclusion criteria have been described previously [21]. The participants were recruited by practitioners performing the screening of potential candidates in two primary health care centers. Some participants were also recruited through dissemination of the study by board advertisements.

Seventy-three enrolled participants were randomized to the Placebo (PLC, *n* = 35) or Moringa (MO, *n* = 38) groups. Sample size calculation and randomization details are provided in [21].

This study followed the principles established in the Declaration of Helsinki and the guidelines of the Spanish law 14/2007 on Biomedical Research. Moreover, the study procedures were approved by the Puerta de Hierro-Majadahonda University Hospital Ethics Committee as well as the Bioethics committee of the Spanish National Research Council (CSIC). Prior to study entry, written informed consent was obtained from all participants.

### 2.3. Intervention

The MO leaves used in the manufacture of the capsule supplement were obtained from an organic cultivar grown in the Mediterranean region of Spain. Nutrient composition and polyphenol content of the dry leaves are presented in Table S1. Further details can be found in [21].

Patients were instructed to take 2 capsules before each main meal (breakfast, lunch and dinner; 2.4 g/day) for 12 weeks. They were asked to make no changes in their diet or lifestyle. Three visits were programmed, at baseline (0 weeks), 6 weeks and 12 weeks. Compliance with capsule intake was good, and additional data have been already published [21].

On each visit, the participants arrived early in the morning in fasting condition. A blood sample was withdrawn from the antecubital vein, which was collected in vacutainer tubes for different biomarker analyses. A first-void urine sample collected at home was also brought on each visit. Participants were given a list of forbidden polyphenol-rich foods and recommended dishes to apply to the previous day’s dinner.

### 2.4. Outcomes

All biomarkers studied in the current work were included as secondary outcomes in the study conception and protocol.

### 2.5. Blood Lipid Profile and Inflammatory Biomarker Analyses

Within two hours from collection, blood was centrifuged at 1300× *g* for 15 min, and several aliquots were kept at −80 °C until analysis. Lipid profile and C-reactive protein (hsCRP) were analyzed in serum of freshly collected samples (Unilabs Laboratory, Madrid, Spain). Inflammatory cytokines (IL-1β, TNF-α, IL-6 and macrophage chemoattractant protein (MCP)-1) and adipokines (leptin and adiponectin) were analyzed at ICTAN laboratory by xMAP Luminex^®^ technology (Luminex Corporation, Austin, TX, USA) with the Human High Sensitivity T cell panel and the Metabolic Hormone magnetic bead panels (Merck Millipore Burlington, MA, USA). The protease inhibitors AEBSF (4-(2-aminoethyl) benzenesulfonyl fluoride hydrochloride; 1 mg/mL final concentration) and dipeptidyl peptidase (DPP)-IV inhibitor (10 µL per 1 mL blood) (Merck KGaA, Darmstadt, Germany) were added to 1 mL of blood before centrifugation and aliquoting for preserving proteins from degradation. The sensitivity (minimum detectable concentration) of these measurements was as follows: IL-1β, 0.14 pg/mL; TNF-α, 0.16 pg/mL; IL-6, 0.11 pg/mL; MCP-1, 14 pg/mL; leptin, 41 pg/mL; adiponectin, 11 pg/mL.

### 2.6. TAC Assessment

The measurement of TAC was performed following the application note for Photochem (Analytikjena, Jena, Germany) for the determination of lipid-soluble antioxidant capacity in blood plasma. In brief, a sample of 200 μL plasma was mixed with 200 μL dH_2_O and 400 μL ethanol. Then, 800 μL hexane was added, the mixture was shaken for 1 min and centrifuged 1000× *g* for 5 min. Then, 200 μL of the lipidic phase was collected and dried under nitrogen flow and stored in the freezer (−80 °C) until analysis. The dried extract was dissolved in 200 μL methanol and centrifuged 5000× *g* 1 min. Measurement was performed in the supernatant following Photochem application (Analytikjena) using an ACL reagent kit containing Trolox as the standard for the calibration curve and a photosensitizer and 120 µL of sample. The pipetted sample volume was used for final TAC calculation with the formula:

TAC = [Quantity (nmol) × Dilution × Trolox Molarity (ng/nmol)]/pipetted volume (µL)

### 2.7. Blood Pressure

Blood pressure was measured with an OMRON M6 device (Omron Healthcare, Kyoto, Japan) twice each visit, and the lowest measure was used in the analysis. Data from several patients at 6 weeks and 12 weeks were missing due to the COVID pandemic and the compulsory distance measures to avoid virus propagation.

### 2.8. Fecal Sample Biomarkers

Frozen fecal samples (−80 °C) were slightly defrosted, and several weighed aliquots were prepared using a scalpel, for different analysis. A 150–200 mg aliquot was prepared for the analysis of calprotectin and secretory immunoglobulin A (sIgA) and kept at −80 °C until analysis. sIgA and calprotectin were analyzed with commercial ELISA kits by Immundiagnostik AG (IDK^®^, Bensheim, Germany) following the manufacturers’ protocols. Fecal sampling prior to analytical procedures was performed with the Stool Sample Preparation System (ref# K 6998SAS, Bensheim, Germany), which allows the extraction from a standardized amount of 15 mg. The analyses were carried out in duplicate. ELISA plates were read on the PowerWawe XS Spectrophotometer (BioTek, Santa Clara, CA, USA) by reading at 450 nm and fitting the standard curve with non-linear 4-parameter regression. Both kits included high and low quality controls. The detection limit of the kits was 6.947 ng/mL and 2.267 ng/mL for sIgA and calprotectin, respectively.

### 2.9. MO Metabolites in Urine Samples

Collected urine samples were centrifuged at 3000 rpm for 5 min, and 1.5 mL supernatant was filtered through a 0.45 μm pore (13 mm filter), vacuum dried and stored at −80 °C until analysis. Dried samples were reconstituted in 0.2 mL ammonium acetate 13 Mm Ph 4/0.1% formic acid in acetonitrile (50:50, *v*/*v*), vortexed for 2 min and sonicated for an additional 10 min. After sonication, samples were vortexed again (2 min) and centrifuged at 10,000× *g* rpm for 5 min at 4 °C. Supernatants were collected and filtered through a Millex-HV13 0.22 μm pore membrane (Millipore Corp., Bedford, MA, USA).

Identification and quantification of glucosinolates and their metabolic derivatives in the urine of participants was performed by ultra-high pressure liquid chromatography coupled to electrospray ionization and a 6460 tandem mass spectrometer with triple quadrupole technology (UHPLC/MS/MS, Agilent Technologies, Waldron, Germany). Chromatographic separation was carried out using a ZORBAX Eclipse Plus C18 column (2.1 × 50 mm^2^, 1.8 μm) with a chromatographic gradient created with the solvents (A) 13 mM ammonium acetate pH 7 and (B) acetonitrile/formic acid (99.99:0.01, *v*/*v*) according to the method specified in [39], which separates intact glucosinolates and indoles, updated for the analysis of the compounds specifically present in the administered matrix. 

### 2.10. Diet and Anthropometry Assessments

A three-day dietary registry form was used for dietary assessment, as published elsewhere [21]. Anthropometrical measurements collected from the participants included weight, height, waist and hip circumferences and bioimpedance analysis (InnerScan BC-545; TANITA, Tokyo, Japan). All measurements were taken barefoot and with standard methods. Height was measured only at the basal visit with a stadiometer (0.5 cm precision) and weight at each visit with the TANITA scale to the nearest 0.1 kg. BMI was calculated as Weight (kg)/Height (m)^2^. Circumferences were measured with an inelastic tape (SECA, precision 0.5 cm) using standard procedures. A dichotomic variable was created and named as Fat_Status based in the fat mass percentage obtained from the bioimpedance analysis and using the upper threshold for normal body fat percentage established for the TANITA bioelectrical impedance device for men (40–59 year, <22% and 60–70 year, <25%) and women (40–59 year, <34% and 60–70 year, <36%) [40]. This variable classified participants as “Normal body fat” or “Above normal body fat”.

Other questionnaires were also filled in by trained nutritionists on each occasion, including the MEDAS questionnaire of adherence to the Mediterranean diet [41] and the Minnesota Leisure Time Physical Activity Questionnaire (MLTFAQ, Spanish version). The latter was administered by the interviewer at the basal visit, and at subsequent visits the volunteers went through their responses and highlighted any differences in their previously recorded activities. For each individual, METs (metabolic standard units) were estimated using the coefficients published in the Compendium of Physical Activities [42] and transformed to kcal/week by considering minutes spent in each physical activity per week, weight and the equivalence 0.0175 kcal/minute/kg.

### 2.11. Statistical Analysis

Prior to data analyses, the Kolmogorov–Smirnov test and box-plot representation was used to check the distribution of the variables. Normalization of several variables was obtained after log transformation, including leptin, adiponectin, calprotectin, sIgA, TAG and VLDL-C.

The effect of the MO supplementation was assessed by comparison of the change score between the groups. 

The rate of change for blood and fecal biomarkers was calculated with the following formula:

[(value 12 weeks − value 0 weeks)/value 0 weeks] × 100

The difference between the groups in the variable’s rate of change was assessed by ANCOVA, with the fixed factors “treatment” and “body fat mass percentage status” and using the basal value as a covariate. In addition, a MIXED linear model with the repeated factor “visit”, the fixed factors “treatment” and “Fat_Status” and the interactions “visit × treatment” and “visit × Fat_Status” was used to compute the effect of the MO food supplement versus PLC along the intervention. For lipid profile variables’ analysis, having a prescription of lipid lowering agents was also used as a dichotomic variable (yes or no) to further correct the model. Similarly, for blood pressure analyses, the confounding factor “antihypertensive agent” (YES/NO) was also included to adjust the model. In addition, two individuals starting anti-hypercholesterolemic drug treatment during the intervention as well as another two starting antihypertensive treatment were excluded from the respective analyses.

In order to assess if the individual characteristics related to the cardiometabolic and inflammatory status influenced the glycemic response to MO supplementation of the patients with prediabetes, a decision tree approach was undertaken. This statistical analysis aimed to classify cases into Respondent (improves HbA1c) or Non-respondent (does not improve HbA1c) based on values of independent (predictor) variables. The tree-based classification model was created using the following as independent variables (potential predictors): sex and Fat_Status (categorical), age, BMI, total cholesterol, HDL-C, VLDL-C, LDL-C, TAG, TAC, systolic pressure, diastolic pressure, leptin, adiponectin, MCP-1, TNF-α, IL1-β, IL-6, calprotectin and sIgA (continuous variables); the nominal variable HbA1c improvement (YES/NO) was used as a dependent variable and CHAID (CHi-square Automatic Interaction Detection) as the growing method. In addition, the correlations between the basal levels of biomarkers and the change in HbA1C during the intervention were also analyzed.

## 3. Results

**Figure 1:**
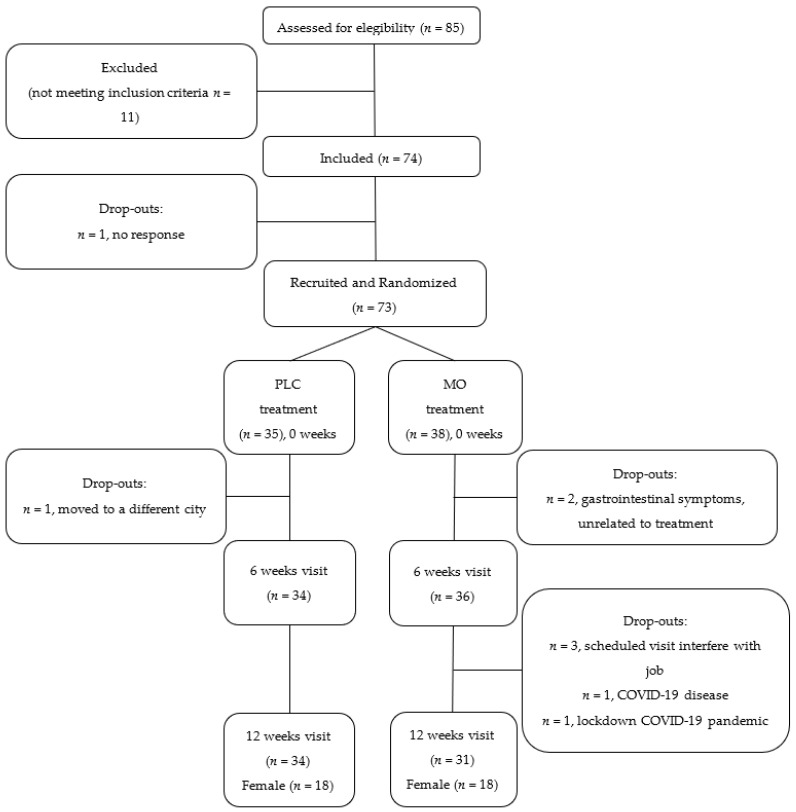
Flow chart of study participants.

Figure 1 presents a flow chart of study participants, showing that 65 participants finished the study, distributed as 34 in PLC (18 female) and 31 in MO (18 female) groups. Table 1 presents their basal characteristics. No differences were found between PLC and MO groups in the proportion of subjects with an excess of body fat, which had a high prevalence in both groups. No differences were found either in the prevalence of medication prescription for cardiovascular risk factors, the MEDAS score or the amount of energy expenditure in leisure time physical activity.

The demographic, anthropometrical and routine biochemistry test results in the study participants, classified according to their fat mass percentage, are presented in Table 2. In addition to the anthopometrical differences, plasma routine biochemical parameters such as those related to glycemia (glucose and HbA1c), TAG, VLDL-C and hsCRP were different between participants with a different Fat_Status classification.

The results showed no significant differences between groups in the change of cytokines, adipokines and hsCRP during the intervention and no significant interaction of time × treatment on these markers’ values (Table 3). 

No significant visit × treatment interactions were observed in blood lipid profile variables or TAC (Table 4), and no differences in the rate of change were observed between the groups.

Two participants that started anti-hypertensive medication during the intervention were excluded from the analysis. A total of 44 valid participants had basal and final measurements of blood pressure taken, and the statistical analysis showed no significant differences in the change score of either SBP or DBP between treatment groups and no significant interaction of visit × treatment was observed during the intervention (Table 5).

No effect of treatment was observed on variables of intestinal health, i.e., calprotectin and sIgA (Table 6). The number of participants with positive values for calprotectin (>100 µg/mL) was not different either between groups at baseline or end of treatment (27 vs. 17% at 0 weeks and 18 vs. 17% at 12 weeks, in PLC and MO, respectively).

**Figure 2:**
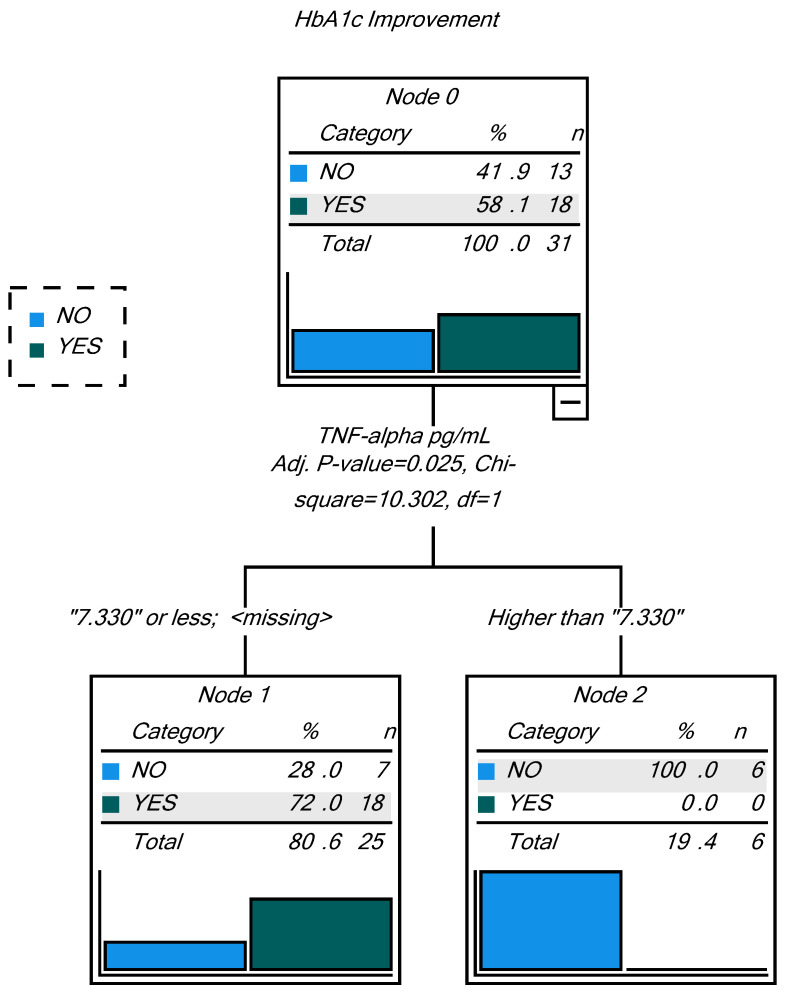
Decision tree on HbA1c improvement as dependent variable. Classification is 77% correct using basal TNF-α as predictor variable.

The decision tree analyses showed that TNF-α plasma concentration significantly contributed to 77% correct classification of participants as respondents or non-respondents to MO supplementation (HBA1c improvement, YES/NO) (Table 7), with a discriminant threshold at 7.330 pg/mL (Figure 2). In addition, a significant correlation was found in the MO supplemented group between the change in HbA1c and the basal TNF-α value (r = 0.361; *p* = 0.050; r = 0.375; *p* = 0.045 in partial correlation with BMI adjustment). On the contrary, in the PLC group, no variable was successful in the decision tree at correctly classifying the subjects that improved HbA1c during the intervention, and no significant correlation was found. 

Regarding the identification of MO metabolites in urine, only in 36% of supplemented participants was glucomoringin detected in urine, while moringin was detected in 71%. No relationship was observed between the presence of these MO characteristic molecules and improvement of HbA1c during the study. Glucomoringin was identified in 30% of the participants that improved HbA1c levels during the intervention and in 46% of the non-respondent participants (Chi^2^ test; *p* = 0.346); for the ITC moringin, these percentages were 71% and 77%, respectively (Chi^2^ test; *p* = 697).

## 4. Discussion

Despite a vast body of evidence on the in vitro antioxidant and anti-inflammatory activities of MO leaf extracts and polyphenolic compound enriched fractions [9,31], as well as evidence from animal studies showing antidiabetic and antihyperlipidemia effects [43], provision of antioxidant stability [44], modification of the expression of enzymes involved in carbohydrate and lipid metabolism [44,45] and of inflammatory markers [29,30,45], the evidence of similar effects and activities in human studies is scarce. This nutritional intervention study was designed to test the hypoglycemic effect of MO dry leaf powder in patients with prediabetes, and a moderate but significant effect was found on glycemia markers [21]. Here, the effects of this intervention on inflammatory and cardiometabolic status markers were assessed as secondary outcomes. The reported results of the inflammation markers measured in serum or plasma (CRP, MCP-1, TNF-α, IL1-β, IL-6) or in fecal samples (calprotectin), the leptin and adiponectin levels, the lipid profile, the plasma antioxidant capacity and the blood pressure showed no significant effects. The dose of MO used, added to individual heterogeneity in the biomarker basal values, might have contributed to the lack of significant effects observed.

There are no clinical trials in the literature studying changes in plasma inflammatory markers following MO leaf intake. Many animal model studies have shown that MO leaf extracts in daily amounts of 200–300 mg/kg BW(body weight) decrease the expression of proinflammatory cytokine genes and the protein levels in different organs [11,30]. However, at higher doses, different results have also been reported. Gao et al. [46], in an experiment with mice, when administering 750 mg/kg BW of an aqueous MO leaf extract for 4 weeks, showed impaired colon intestinal barrier and increased inflammatory response as suggested by increased serum LPS and expression of pro-inflammatory cytokines in the colon and liver, although with inconsistent cytokine expression levels in the ileum. This concurred with alterations in bacterial groups in the cecum samples. In humans, the effect of MO on inflammatory conditions has been tested in individuals with gingival inflammation. A cross-over study including 20 subjects with mild to moderate gingivitis showed a greater reduction in mean gingival index scores and plaque scores with the MO-based dentifrice compared to a miswak-based dentifrice [47]. In addition, another study with MO leaf extract-based lozenges reduced inflammation and gingivitis in smokers [48]. In support of these findings, a model of periodontitis as a chronic inflammatory disease proved that MO leaf aqueous extract at 0.5 and 1 g/kg BW for 30 days provides anti-periodontitis activity in rats. The treatment significantly decreased serum TNF-α, IL-1β, and IL-6 compared with the control group, whereas IL-1Ra and IL-10 were increased and alveolar bone resorption was significantly reduced [49]. Despite the fact that no significant effects of MO consumption were found on inflammatory markers in the current study, no definitive conclusions can be drawn regarding the anti-inflammatory potential of MO supplementation because of the variable inflammatory status of the participating subjects. A population with homogeneously high inflammatory markers might have facilitated finding significant anti-inflammatory effects. This, together with the fact that the administered dose was in the low range of those used among the published studies might explain why the anti-inflammatory activity of MO was not observed in the current study. 

Two clinical trials have assessed the results of MO leaf tablets or powder consumption on the lipid profile. The first one included 35 hyperlipidemic individuals and showed that the consumption of 4.6 g tablets of MO leaves for 50 days resulted in a small (1.6%) but significant (*p* < 0.05) decrease in total cholesterol and in the total cholesterol/HDL-C ratio (*p* < 0.001), with a non-significant increase in HDL-C (6.3%) and unchanged TAG, LDL-C and VLDL-C [36]. The second study by Kumari et al. [17] included non-insulin dependent diabetic patients (23 in the experimental group and 9 in the control group) and showed a significant reduction in total cholesterol, LDL-C and VLDL-C after a 40-day intervention with 8 g MO leaf powder per day, without a significant change in HDL-C, which increased by 9%. The difference with the lack of effect observed in the current study might be due to the lower dose used here and the difference in the mean initial values of total cholesterol between studies, which were 198 ± 32 mg/dL in the experimental group of the current study and 261 ± 20 mg/dL in the MO supplemented subjects with diabetes studied by Kumari et al. [17]. In this sense, it is worth mentioning that the subjects with prediabetes studied in the current clinical trial had variable nutritional status as assessed by BMI and body composition measures. As a result, differences were found in plasma routine biochemical parameters such as those related to glycemia (glucose and HbA1c), lipemia (TAG, VLDL-C) and inflammation (CRP) between participants with normal and above-normal fat percentage, which supports the need to include the categorical variable of fat status (based in excessive body fat percentage relative to normal values) as a potential confounding factor in this type of studies.

The radical scavenging and antioxidant activity of MO leaves has been amply demonstrated in vitro using different solvent extractions [50]. However, evidence from human studies is scarce. In a study by Ngamukote et al. [51], 20 healthy participants were assigned to receive a single dose of either 200 mL of warm water or 200 mL of MO aqueous leaf extract (500 mg). The increase in ferric reducing ability of plasma (FRAP) and decrease in malondialdehyde (MDA), the main product of lipid peroxidation, were significant 30 min after ingestion of the MO extract. In addition, a negative correlation was observed between both parameters. This study suggests the potential to beneficially modify plasma antioxidant status; however, it provides limited evidence due to the small sample size and does not provide results for long-term consumption. Another study supplemented MO powder (7 g) within daily menus for 3 months in postmenopausal women, showing a significant improvement in the blood antioxidant levels, including serum retinol, ascorbic acid, glutathione peroxidase and superoxide dismutase, whereas MDA was decreased [19]. The participating women were from the Indian state of Punjab, where MO is a traditionally consumed food. Depending on the dryness degree of the leaf powder, the concentration and actual ingested amount of bioactive compounds can vary; however, the dose used in the study by Kushwaha et al. was larger than the one in our study, and this might explain why we were not able to observe an improvement in the plasma antioxidant capacity. The method we used to measure plasma antioxidant capacity analyzed exclusively the circulating antioxidants in the plasma lipophilic fraction, which was chosen to exclude the effect of uric acid, known as the main contributor to plasma total antioxidant capacity as measured using various methods i.e., FRAP [52]. We did not measure MDA, and we cannot exclude that MO exerted an effect on this marker or the enzyme-dependent antioxidant systems.

Another study by Taweerutchana et al. [20], testing the hypoglycemic activity in therapy-naïve diabetic patients failed to find a significant effect of MO leaf powder compared to the placebo as evaluated through fasting and postprandial glucose levels monitored with glucometers. According to the authors, this might be related to an insufficient amount of bioactive compounds, like moringin or chlorogenic acid, in the 4 g of MO dry leaf powder provided per day. In addition, in that study, the 28-day duration of the intervention might have been too short. Interestingly, despite no change in antihypertensive agents, the MO leaf group had a reduction of SBP and DBP by 5 mmHg compared to the baseline, whereas the placebo group showed an increase in blood pressure by 2 mmHg; however, these differences had no statistical significance. Similarly, in the current study, blood pressure did not show significant changes following MO supplementation compared to the PLC group; however, due to missing data in the final visit, the sample size for this parameter was smaller than for the rest of the parameters. This lack of effect contrasts with previous observations in animal models [34,35] and human studies [37,53] that substantiate the use of the plant in traditional medicine. Evidence has been published of the capacity of MO to lower 2 h postprandial blood pressure in healthy participants that consumed 120 g of cooked MO for a week [37]. However, long-term randomized placebo-controlled studies have not been published. Thus, a bigger study in therapy-naïve hypertensive subjects with long-term supplementation is warranted.

Despite the lack of significant changes in all secondary outcomes reported in this study, an interesting finding was the difference in baseline TNF-α between participants improving their glucose control and those who did not improve with MO supplementation. In the MO supplemented group, 58% of patients improved their HbA1c values during the intervention, compared to 38% in the PLC group [21]. One hundred percent of participants were correctly classified as respondents based on basal TNF-α ≤ 7.330 pg/mL. However, although the threshold of 7.330 pg/mL for basal value TNF-α is the best predictor of HbA1c response, 28% of those subjects with values under this threshold will still be non-respondents, probably because of other factors not measured in this study, which might include genetic polymorphisms and epigenetic regulation of genes involved in carbohydrate and lipid metabolism [54] or microbiome differences between subjects [55,56]. On the other hand, considering the predictive character of this test, this screening method would facilitate the identification of 19% of subjects with prediabetes that would not respond due to high basal TNF-α values, under the conditions used for supplementation in this study. So far, the findings indicate that the presence of chronic low-grade inflammation hinders the benefits of low-dose MO supplementation on glycemic control. Thus, it is possible to speculate that higher doses might work more efficiently to improve both glycemic control and the inflammatory cytokines. Further studies with higher doses adapted to the subjects’ inflammatory condition would be necessary.

This study is limited by the low dose used compared to other human studies and the mild baseline metabolic damage observed in the participating subjects as a group, although individual characteristics showed heterogeneity as observed in variable fat excess and differences in baseline laboratory values. The study had several advantages, such as a higher sample size than previously published studies, the blinded design of the intervention and the amount of different parameters measured, which allowed the screening of baseline characteristics to point to TNF-α as a good predictor of the participants’ glycemic response to MO. 

In conclusion, although this study with MO supplementation improved the glycemic control of participants with prediabetes, as previously published [21], no further effects were evidenced in inflammatory and cardiometabolic markers. The relationship observed between higher basal TNF-α values and failure to improve glycemic control suggests that doses higher than 2.4 g per day might be necessary to increase the number of subjects with a favorable response in all of the biomarkers studied.

## Figures and Tables

**Table 1:** Basal characteristics and medication prescribed in the study participants.

	PLC (*n* = 34)	MO (*n* = 31)	*p* ^#^
BMI < 25, *n* (%)	6 (18)	4 (13)	0.561
25–29.9, *n* (%)	17 (50)	13 (42)	
≥30, *n* (%)	11 (32)	14 (45)	
Fat_Status			0.683
Normal, *n* (%)	8 (23.5)	6 (19.4)	
Above normal, *n* (%)	26 (76.5)	25 (80.6)	
Body fat excess (%) ^a^	5.1 ± 6.2	5.7 ± 4.4	0.327
Lipid lowering agents, *n* (%)	8 (23.5)	10 (32.3)	0.432
Antihypertensive agents, *n* (%)	9 (26.5)	7 (22.6)	0.716
MEDAS score	9.5 ± 2.5	10.2 ± 1.8	0.208
Physical activity (kcal/week)	4591 ± 2831	3890 ± 2176	0.279

^#^ Chi^2^ or Student’s *t* test (independent samples). ^a^ Body fat excess is calculated as body fat percentage minus the high threshold of the normal body fat percentage range for men and women according to Gallagher et al. (2000) [40] for the corresponding age group (40–59 year or 60–70 year). PLC: placebo; MO: *Moringa oleifera* Lam.

**Table 2:** Basal characteristics of the participants with prediabetes according to their body fat percentage.

	Normal Body Fat % (*n* = 14)	High Body Fat % (*n* = 51)	*p*
Male:Female ^#^, *n* (%)	7:7 (50:50)	22:29 (43:57)	0.647
Age (year)	53.3 ± 11.4	56.9 ± 9.6	0.116
BMI	24.3 ± 1.9	30.2 ± 3.3	<0.001
<25 ^#^ (*n*)	10	0	<0.001
WC (men)	87.6 ± 5.3	101.8 ± 9.7	<0.001
(women)	84.7 ± 5.8	94.9 ± 9.7	<0.001
Glucose (mg/mL)	98.3 ± 9.6	105.3 ± 14.1	0.042
HbA1c (%)	5.7 ± 0.3	5.9 ± 0.3	0.014
Uric acid (mg/dL)	5.4 ± 0.7	5.6 ± 1.4	0.246
GOT (UI/L) ^Φ^	21.50 (17.75–25.50)	22.00 (19.00–27.00)	0.835
GPT (UI/L) ^Φ^	20.50 (15.00–31.50)	26.00 (21.00–33.00)	0.089
GGT (UI/L) ^Φ^	20.00 (14.75–29.75)	24.00 (19.00–36.00)	0.177
Total Cholesterol (mg/dL)	207.0 ± 38.1	202.1 ± 33.7	0.327
TAG (mg/dL)	83.6 ± 18.9	111.8 ± 47.3	0.003
HDL-C (mg/dL)	58.2 ± 12.3	58.1 ± 13.3	0.488
LDL-C (mg/dL)	131.9 ± 34.2	121.7 ± 31.5	0.147
VLDL-C (mg/dL)	16.9 ± 3.9	22.4 ± 9.4	0.004
hsCRP (mg/dL) ^Φ^	0.03 (0.006–0.09)	0.16 (0.07–0.51)	0.001

Data are Mean ± SD or Median (IQR, interquartile range). Mean values between groups were compared by independent sample *t* test, except for the variables specified with the symbols. ^#^ Chi^2^ test was used for categorical variables and ^Φ^ Mann-Whitney U test was used for variables not fitting normal distribution. The log-transformed variables were used for group comparison in the case of HbA1c, total cholesterol, TAG and VLDL-C. WC, waist circumference; TAG, Triacylglycerides. GGT: Gamma-glutamyl Transferase.

**Table 3:** Inflammatory markers and adipokines in patients with prediabetes of the PLC and MO groups during the intervention.

		0 Weeks	6 Weeks	12 Weeks	MIXED Model p ^#^	Rate of Change ^a^ 0 Weeks–12 Weeks
MCP-1 (pg/mL)	PLC	67, 82 ± 57	69, 77 ± 42	69, 77 ± 36	0.328	0.063 ± 0.418
	MO	63, 71 ± 35	70, 64 ± 24	57, 69 ± 45		−0.002 ± 0.312
						NS
TNF-α (pg/mL)	PLC	6.0, 7.4 ± 3.7	6.4, 7.1 ± 3.5	5.5, 6.7 ± 3.1	0.291	−0.057 ± 0.232
	MO	5.7, 6.4 ± 2.4	6.5, 6.8 ± 2.8	5.5, 6.0 ± 2.3		−0.034 ± 0.279
						NS
IL-6 (pg/mL)	PLC	1.8, 2.6 ± 3.0	1.7, 3.7 ± 7.3	1.8, 2.3 ± 2.1	0.607	−0.012 ± 0.599
	MO	1.3, 3.6 ± 7.9	1.7, 4.9 ± 11.5	1.2, 3.2 ± 9.1		−0.158 ± 0.368
						NS
IL-1β (pg/mL)	PLC	1.4, 1.7 ± 1.1	1.3, 1.7 ± 1.0	1.3, 1.6 ± 0.9	0.908	−0.008 ± 0.372
	MO	1.4, 1.5 ± 0.8	1.3, 1.5 ± 0.8	1.1, 1.3 ± 0.6		−0.061 ± 0.328
						NS
hsCRP (pg/mL)	PLC	0.15, 0.40 ± 0.64	0.11, 0.31 ± 0.67	0.12, 0.30 ± 0.50	0.359	0.457 ± 2.457
	MO	0.10, 0.23 ± 0.41	0.11, 0.19 ± 0.21	0.11, 0.19 ± 0.22		1.189 ± 3.423
						NS
Leptin (ng/mL)	PLC	5.63, 7.59 ± 7.97	6.61, 7.75 ± 8.29	5.66, 6.72 ± 5.45	0.343	0.072 ± 0.454
	MO	6.48, 7.15 ± 4.68	8.35, 7.96 ± 4.73	5.87, 6.99 ± 4.95		−0.017 ± 0.387
						NS
Adiponectin (µg/mL)	PLC	23.1, 27.7 ± 21.7	21.09, 24.5 ± 16.6	15.42, 18.5 ± 12.0	0.871	−0.242 ± 0.338
	MO	23.4, 26.5 ± 16.1	15.91, 23.2 ± 16.4	15.60, 18.8 ± 10.9		−0.262 ± 0.252
						NS

Median, Mean ± SD. MIXED linear model with the repeated factor “visit” and the fixed factors “treatment” and “Fat_Status” and the interaction “visit × treatment” and “Fat_Status × treatment”; p ^#^ corresponds to “visit × treatment”. ^a^ ANCOVA with the fixed factors “treatment” and “Fat_Status” and using the basal value as covariate; NS, not significant. PLC: placebo; MO: *Moringa oleifera* Lam.; MCP: macrophage chemoattractant protein.

**Table 4:** Serum lipid profile and plasma TAC in patients with prediabetes of the PLC and MO groups during the intervention.

		0 Weeks	6 Weeks	12 Weeks	MIXED Model p ^#^	Rate of Change 0 Weeks–12 Weeks ^a^
Total Cholesterol (mg/dL)	PLC	206.4 ± 36.4	207.7 ± 36.0	211.2 ± 34.6	0.494	0.033 ± 0.122
	MO	197.9 ± 31.5	208.6 ± 29.1	203.1 ± 35.0		0.011 ± 0.123
						NS
TAG (mg/dL)	PLC	102.2 ± 46.5	106.7 ± 43.2	113.0 ± 51.2	0.824	0.143 ± 0.352
	MO	110.2 ± 43.3	115.82 ± 49.5	121.8 ± 72.8		0.110 ± 0.297
						NS
HDL-C (mg/dL)	PLC	57.8 ± 12.0	59.6 ± 13.4	61.8 ± 15.0	0.608	0.073 ± 0.157
	MO	57.8 ± 14.3	59.1 ± 14.7	58.9 ± 14.9		0.026 ± 0.176
						NS
LDL-C (mg/dL)	PLC	128.1 ± 34.4	126.8 ± 33.2	126.7 ± 34.5	0.307	0.001 ± 0.155
	MO	118.1 ± 27.9	126.4 ± 27.4	119.8 ± 30.0		0.000 ± 0.184
						NS
VLDL-C (mg/dL)	PLC	20.4 ± 9.3	21.3 ± 8.6	22.7 ± 10.1	0.776	0.145 ± 0.349
	MO	22.1 ± 8.5	23.1 ± 9.9	24.4 ± 14.6		0.096 ± 0.296
						NS
TAC	PLC	1.34 ± 0.32	1.29 ± 0.37	1.30 ± 0.45	0.134	−0.01 ± 0.32
	MO	1.22 ± 0.42	1.27 ± 0.39	1.09 ± 0.44		0.03 ± 0.57
						NS

Mean ± SD. MIXED linear model with the repeated factor “visit” and the fixed factors “treatment”, “Fat_Status” and “anti-hypercholesterolemic treatment” and the interaction “visit × treatment” and “visit × Fat_status”; p ^#^ corresponds to “visit × treatment”. ^a^ ANCOVA with the fixed factor “treatment”, the confounding factors “Fat_Status” and “anti-hypercholesterolemic treatment” and the basal value as covariate. TAG, triacylglycerides; NS, not significant. PLC: placebo; MO: *Moringa oleifera* Lam.

**Table 5:** Systolic and diastolic blood pressure in patients with prediabetes of the PLC and MO groups during the intervention.

		0 Weeks PLC, *n* = 34; MO, *n* = 29	6 Weeks PLC, *n* = 29; MO, *n* = 22	12 Weeks PLC, *n* = 26; MO, *n* = 18	MIXED Model p ^#^	Rate of Change 0 Weeks–12 Weeks ^a^ PLC, *n* = 26; MO, *n* = 18.
SBP (mmHg)	PLC	129 ± 15	127 ± 187	128 ± 16	0.807	−0.005 ± 0.099
	MO	129 ± 15	125 ± 11	126 ± 11		−0.011 ± 0.077
						NS
DBP (mmHg)	PLC	79 ± 9	78 ± 11	81 ± 11	0.441	0.007 ± 0.083
	MO	80 ± 9	76 ± 8	77 ± 8		−0.031 ± 0.063
						NS

Mean ± SD. MIXED linear model with the repeated factor “visit” and the fixed factors “treatment”, “Fat_Status” and “anti-hypertensive treatment” and the interaction “visit × treatment” and “visit × Fat_status”; p ^#^ corresponds to “visit × treatment”. ^a^ ANCOVA with the fixed factor “treatment”, the confounding factors “Fat_Status” and “anti-hypertensive treatment” and the basal value as covariate. SBP, systolic blood pressure; DBP, diastolic blood pressure; NS, not significant. PLC: placebo; MO: *Moringa oleifera* Lam.

**Table 6:** Intestinal health markers in fecal samples of patients with prediabetes of the PLC and MO groups during the intervention.

		0 Weeks	6 Weeks	12 Weeks	MIXED Model p ^#^	Rate of Change 0 Weeks–12 Weeks ^a^
Calprotectin (µg/mL)	PLC	25; 73 ± 95	-	30; 58 ± 70	0.851	0.0; 0.5 ± 1.6
	MO	32; 58 ± 69	-	32; 59 ± 63		0.1; 0.6 ± 1.6
						NS
sIgA (µg/mL)	PLC	1448; 2017 ± 1856	-	1402; 2208 ± 2437	0.941	−0.1; 1.5 ± 5.8
	MO	1343; 1663 ± 1478	-	1245; 2187 ± 2154		−0.2; 1.0 ± 3.5
						NS

Median, Mean ± SD. MIXED linear model with the repeated factor “visit” and the fixed factors “treatment” and “Fat_Status” and the interaction “visit × treatment” and “Fat_Status × treatment”; p ^#^ corresponds to “visit × treatment”. ^a^ ANCOVA with the fixed factor “treatment”, “Fat_Status” as confounder and the basal value as covariate; NS, not significant. PLC: placebo; MO: *Moringa oleifera* Lam.

**Table 7:** Classification of participants in the decision tree analysis based in the threshold value of TNF-α.

Observed	Predicted		
	NO	YES	Percent Correct
NO	6	7	46.2%
YES	0	18	100.0%
Overall Percentage	19.4%	80.6%	77.4%

Growing Method: CHAID. Dependent Variable: HbA1c improvement.

## Data Availability

The data presented in this study are available on request from the corresponding author, due to privacy restriction. The study is registered in www.ClinicalTrials.gov (Identifier: NCT04734132; accessed on 4 May 2022).

## Supplementary Materials

The following are available online at https://www.mdpi.com/article/10.3390/nu14091937/s1, Table S1: Nutrient and polyphenol content in the MO (*Moringa oleifera* Lam.) dry leaf powder.
