# Supplementary material for: Effects of Moringa oleifera Lam. Supplementation on Inflammatory and Cardiometabolic Markers in Subjects with Prediabetes
Source: Nutrients. 2022 May 5;14(9):1937. doi: 10.3390/nu14091937 (PMC9099674; doi:10.3390/nu14091937)
Supplement: Supplementary file 1 [file nutrients-14-01937-s001.zip › nutrients-1696561-supplementary.pdf]

Table S1. Nutrient and polyphenol content in the MO(*Moringa oleifera* Lam.) dry leaf powder.

**NUTRITIONAL ANALYSIS**	
Energy (kcal/100 g)	350
Energy (kj/100 g)	1465
Fat (%)	4.66
Of which: saturated (%)	0.70
Carbohydrates (%)	49.27
Of which: sugars (%)	0.05
Proteins (%)	27.78
Salt (%)	0.71
**CHEMICAL ANALYSIS**	
Calcium (mg/100g)	4854
Potassium (mg/100g)	2761
Magnesium (mg/100g)	543
Sodium (mg/100g)	541
Phosphorus (mg/100g)	46
Iron (mg/100g)	21
Zinc (mg/100g)	8
Manganese (mg/100g)	5
Total polyphenols (ppm)	2300
